# The patient with patellar instability has a stenotic intercondylar notch and a thin anterior cruciate ligament: a retrospective comparative study

**DOI:** 10.1186/s13018-023-03632-9

**Published:** 2023-02-27

**Authors:** Kuo Hao, Yingzhen Niu, Lingce Kong, Fei Wang

**Affiliations:** Department of Orthopaedic Surgery, Third Hospital of Hebei Medical University, Shijiazhuang, 050051 Hebei China

**Keywords:** Patellar instability, Anterior cruciate ligament, ACL reconstruction, Intercondylar notch, Morphology

## Abstract

**Background:**

Patellar instability (PI) usually combines with morphological abnormalities of femoral condyles that may affect the morphology of the intercondylar notch and anterior cruciate ligament (ACL), which are important in individualized ACL reconstruction. This study aimed to investigate the morphological characteristics of the intercondylar notch and ACL in patients with PI.

**Methods:**

80 patients with PI and 160 age- and gender-matched controls from January 2014 to June 2022 were studied. Morphological measurements of the femoral condyles included intercondylar notch height, notch width, medial condylar width, lateral condylar width, bicondylar width, notch width index, notch angle, lateral femoral condyle ratio (LFCR), condyle flexion angle, and posterior tibial slope. Morphological measurements of the ACL included ACL length, inclination angle, and ACL size. The measurements were compared between PI and control groups, and between males and females in PI group. The independent samples *t*-test was performed to examine differences in continuous variables. The chi-square test was used for comparing categorical variables.

**Results:**

The intercondylar notch width, bicondylar width, notch width index, and notch angle were significantly smaller, while the LFCR was significantly larger in PI group than those of control group (*p* < 0.05). The ACL thickness (0.70 ± 0.16 cm vs 0.80 ± 0.21 cm, *p* = 0.023) and width (0.54 ± 0.14 cm vs 0.60 ± 0.13 cm, *p* = 0.029) were significantly smaller in PI group. The notch width was significantly smaller in female patients than males in PI group, but no significant difference was observed in the notch width index and notch angle (*p* > 0.05). No sex difference related to the morphology of the ACL was found.

**Conclusions:**

The patient with PI had a stenotic intercondylar notch and a thin ACL. No significant sex difference in the intercondylar notch stenosis and ACL size was observed. The morphology of the intercondylar notch and ACL should be taken into consideration when planning individualized ACL reconstruction in the presence of PI.

## Background

Anterior cruciate ligament (ACL) is the most vulnerable ligament within the knee [1]. Reconstruction of the ACL has become a common and effective procedure throughout the world, showing great progresses and breakthroughs in the treatment of ACL injuries. It is important for surgeons to fully understand and restore the normal anatomy of the ACL during reconstruction. Therefore, the concept of “individualized ACL reconstruction” proposed by Fu [2] is becoming increasingly popular, with the goal of restoring native dimensions, collagen orientations, and insertion sites of the ACL to restore native knee biomechanics and improve patient outcomes [3].

As a part of this concept, preoperative planning in ACL reconstruction is essential based on each patient’s native anatomy to determine single-bundle versus double-bundle ACL reconstruction, graft type and size, tibial and femoral insertion site size, and fixation method [4]. The morphology of the femoral intercondylar notch, which houses the ACL as it travels between the femoral and tibial insertions, has gained particular attention [5]. Although the relationship between the morphology of the intercondylar notch and ACL injury remains controversial, many recent studies have emphasized that the narrow intercondylar notch was a predictive risk factor for ACL injuries for both females and males, children and adults, athletes and non-athletes [6–9]. In addition, comparisons between ACL injured and non-injured patients demonstrated that patients with ACL injuries had significantly smaller intercondylar notch volumes [10, 11]. The ACL extends over the inner side of the lateral femoral condylar during flexion, and may impinge on it when subjected to an anterior shear force or tibial rotation, which may lead to ACL injuries [10]. Intercondylar notch can be used as reference in determining the graft size and type during ACL reconstruction [12].

Patellar instability (PI) is a common and debilitating condition that typically occurs in children and adolescents, which is multifactorial and usually combines with morphological abnormalities of femoral condyles that may affect the morphology of the intercondylar notch and ACL [13–15]. Trochlear dysplasia was associated with dysplastic and short lateral posterior femoral condyle in patients with PI [16, 17]. Patients with trochlear dysplasia had smaller lateral posterior condyles and bigger medial posterior condyles compared with those without trochlear dysplasia [18]. Trochlear dysplasia could also extend to the distal intercondylar notch [19]. In addition, increased femoral torsion could affect stability of the patellofemoral joint and the morphology of the femoral condyle [20]. However, the morphology of the intercondylar notch and ACL has not been widely investigated in the presence of PI, which could play an important role in individualized ACL reconstruction.

The aim of this study was to compare the morphology of the intercondylar notch and ACL obtained from magnetic resonance image (MRI) between patients with and without PI, and between males and females with PI. It was hypothesized that the intercondylar notch was narrower, and the ACL was thinner in patients with PI than that of controls. This study could provide useful reference information for individualized surgical planning of ACL reconstruction based on specific morphology of the intercondylar notch and ACL, which can be helpful for surgeons to perform a safe and accurate ACL reconstruction for patients with PI.

## Methods

### Patient selection

**Fig. 1:**
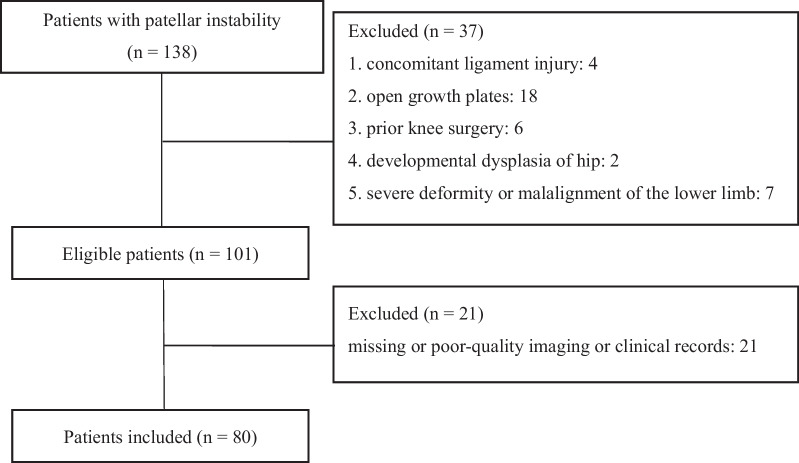
Flowchart of the patient selection in patellar instability group

This retrospective morphological study was approved by the Ethics Committee of our hospital, and the written informed consent was obtained from all included patients. The medical records of the patients with PI between January 2014 and June 2022 at the authors’ institution were identified and reviewed. The inclusion criteria were patients with recurrent, unilateral PI, and skeletal maturity. The recurrent PI was defined as (1) more than one episode of patellar dislocation, or (2) a history of patellar dislocation with symptoms of PI (pain, subluxation, or both), or the positive patellar apprehension sign, for more than 3 months. Patients who met the following criteria were excluded: concomitant ligament injury, open growth plates, prior knee surgery, underlying developmental dysplasia of hip, fracture around the knee, severe deformity or malalignment of the lower limb (valgus or varus > 10°), bony spur formation into the intercondylar notch confirmed by computed tomography or MRI, general joint laxity, rheumatoid arthritis, and severe osteoarthritis with cartilage degeneration > 2 grade according to Kellgren–Lawrence grade. Patients with missing or poor-quality imaging or clinical records were excluded as well. The flowchart of the patient selection in PI group is shown in Fig. 1.

A control group was selected in a 1:2 ratio from skeletal mature patients without PI matched by age, sex, height, and weight during the same time period for comparison. The control group included patients who received MRI for knee complaints other than PI, and showed either isolated meniscal injury or normal anatomy.

By the criteria mentioned above, 80 patients from 138 consecutive patients were enrolled in PI group, and 160 patients were enrolled in control group. Demographic data, including age, sex, height, weight, and laterality, were collected from all patients.

### MRI and radiography

All included patients were examined using the Philips Achieva 1.5-T MRI system (Philips Medical Systems). The patient was in supine position, with the knee in extended position at 10° of flexion, and the lower limb slightly external. The sagittal, coronal and axial sequences with a spin echo T1-weighted image (T1WI), a gradient echo sequence T2 weighted image (T2WI), and a fast spin echo pressure lip sequence, were performed routinely in the protocol. Slice thickness was 3 mm for each plane with a gap of 0.6 mm. The matrix size was 224 × 352 pixels, and the field of view was 16 cm.

True or nearly true lateral radiographs with the overlap between the posterior halves of the medial and lateral condyles were taken at 30° of knee flexion, which included the lateral surface of the patella, and at least the area between half of the femoral shaft and half of the tibial shaft.

### Measurements

All MRI scans and radiographs were reviewed and evaluated carefully by two skilled and independent researchers using RadiAnt-DICOM software (Medixant Ltd., Poznan, Poland), which has an accuracy of 0.01 mm for distance and 0.01° for angles. All of the images were evaluated in a random order. Two researchers were unaware of the patient information, grouping, study purpose and hypothesis. The average measurements were used for final analyses to minimize the measurement error. Any disagreement was resolved through a discussion with another senior researcher until the consensus was reached.

The reliability of all measurements was evaluated using intraclass correlation coefficient (ICC) values with 95% confidence intervals. All measurements were performed by two independent researchers to ensure interobserver reliability. To assess intraobserver reliability, one researcher repeated all measurements with an interval of three weeks. The ICC values ≥ 0.8 were considered as good, ≥ 0.9 were considered as excellent [21].

### Morphology of the femoral condyle

**Fig. 2:**
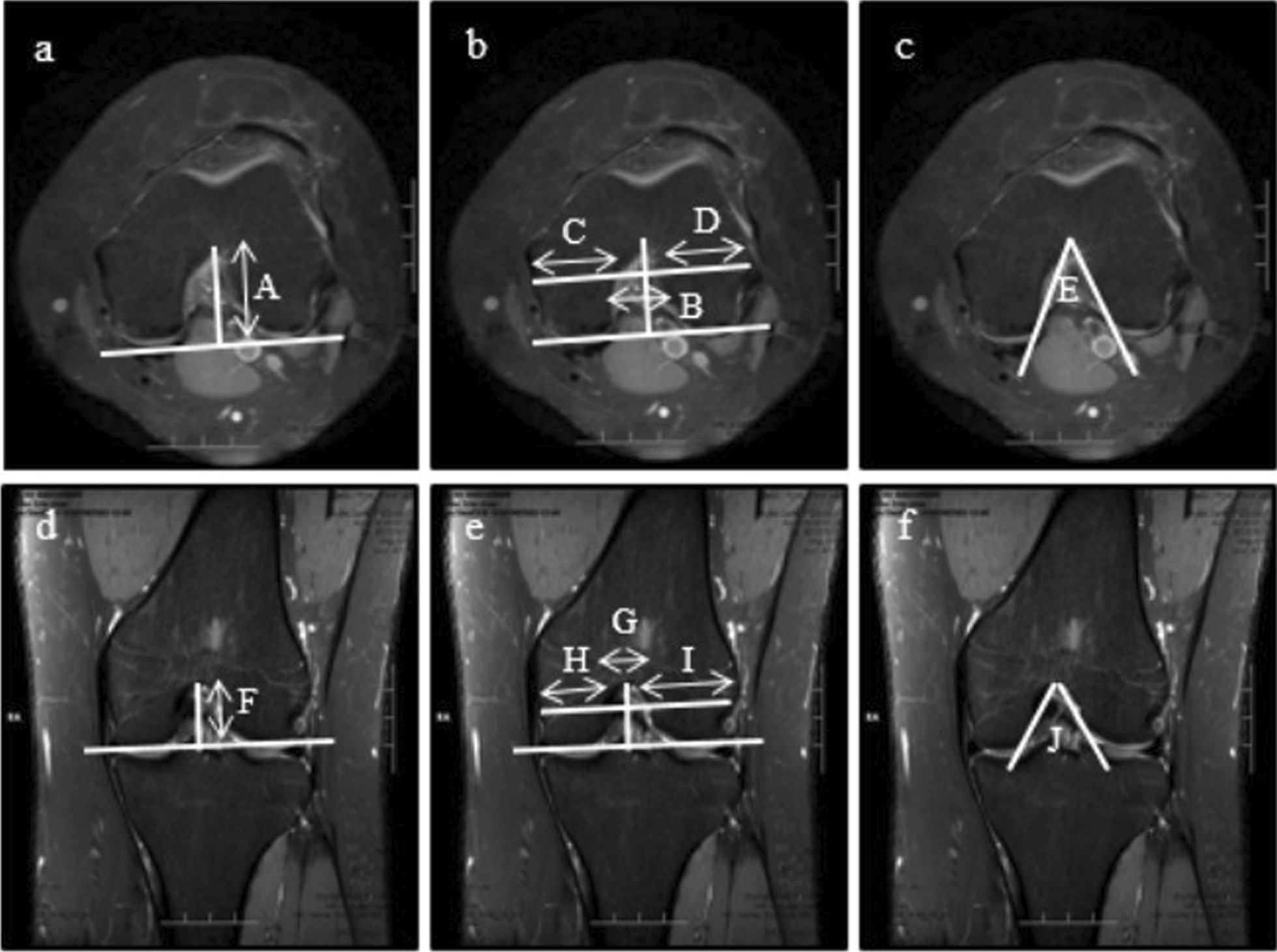
Measurements of the femoral condyles in the axial **a**–**c** and coronal **d–f** planes. The intercondylar notch height **A**, **F** was the distance from the top of the intercondylar notch to the posterior condylar line. The intercondylar notch width **B**, **G** was measured at the anterior third of the notch height. At the same level, medial condylar width **C**, **H** and lateral condylar width **D**, **I** were measured. The notch angle **E**, **J** was formed between the two tangential lines of the entrance of the medial and lateral femoral condyles from the top of the intercondylar notch

Morphological measurements in MRI included intercondylar notch height, notch width, medial condylar width, lateral condylar width, bicondylar width, notch width index, and notch angle in axial and coronal planes. On the axial view, the slice with the deepest popliteal groove was chosen for femoral condyle measurements, as described by Fan et al. [21] and Huang et al. [22]. A posterior condylar line which connected the posterior margins of the medial and lateral femoral condyles was determined. The intercondylar notch height was defined as the distance between the top of the intercondylar notch and the posterior condylar line (Fig. 2a). The notch width was measured at the anterior third of the notch height, which was defined as the distance between medial and lateral edges of the intercondylar notch (Fig. 2b). At the same level, medial condylar width and lateral condylar width were measured (Fig. 2b). The bicondylar width was the sum of the medial condylar width, notch width, and lateral condylar width. The notch width index was the ratio of the notch width to the bicondylar width. The notch angle was defined as the angle between the two tangential lines of the entrance of the medial and lateral femoral condyles from the top of the intercondylar notch (Fig. 2c). On the coronal view, the slice with the highest tibial spine was chosen for measurements, with the same methods used in the axial plane (Fig. 2d–f).

**Fig. 3:**
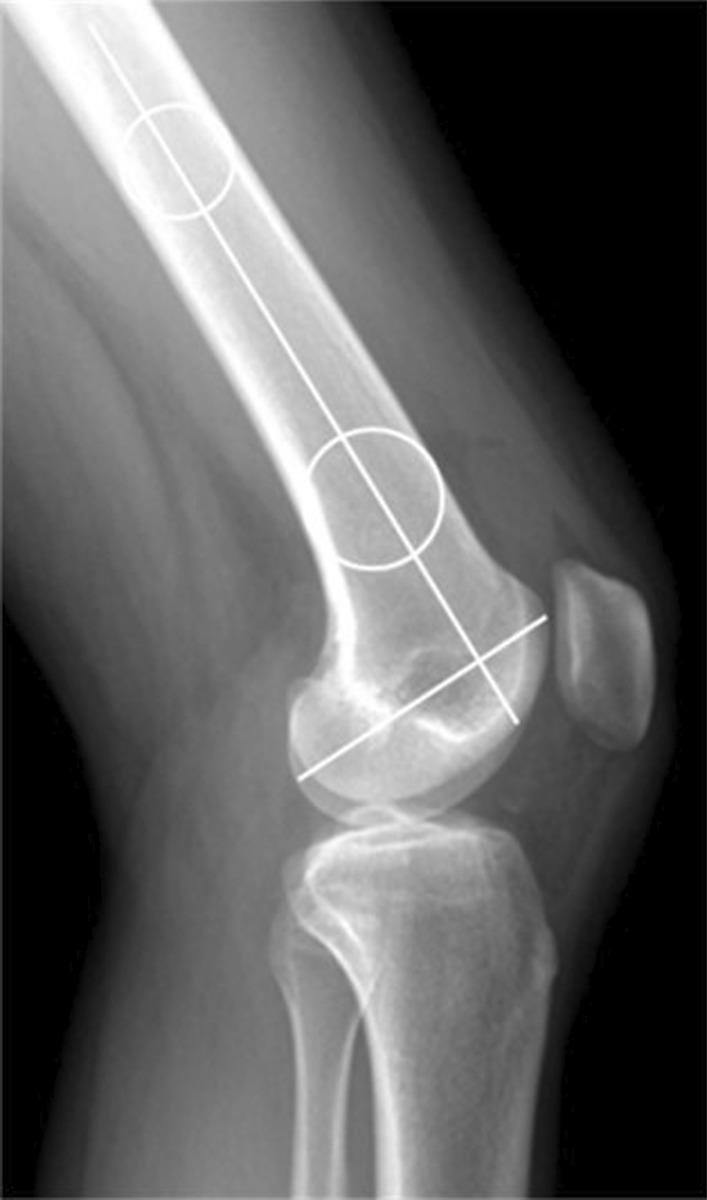
Measurements of the LFCR and CFA. The LFCR was defined as the ratio of the distance from the intersection of the long axis of the distal femur and the axis of the lateral femoral condyle to the most posterior point of the lateral condyle, to the total anteroposterior distance of the lateral condyle. The CFA was defined as the angle between the long axis of the distal femur and the axis of the lateral femoral condyle. *LFCR* Lateral femoral condyle ratio, *CFA* Condyle flexion angle

**Fig. 4:**
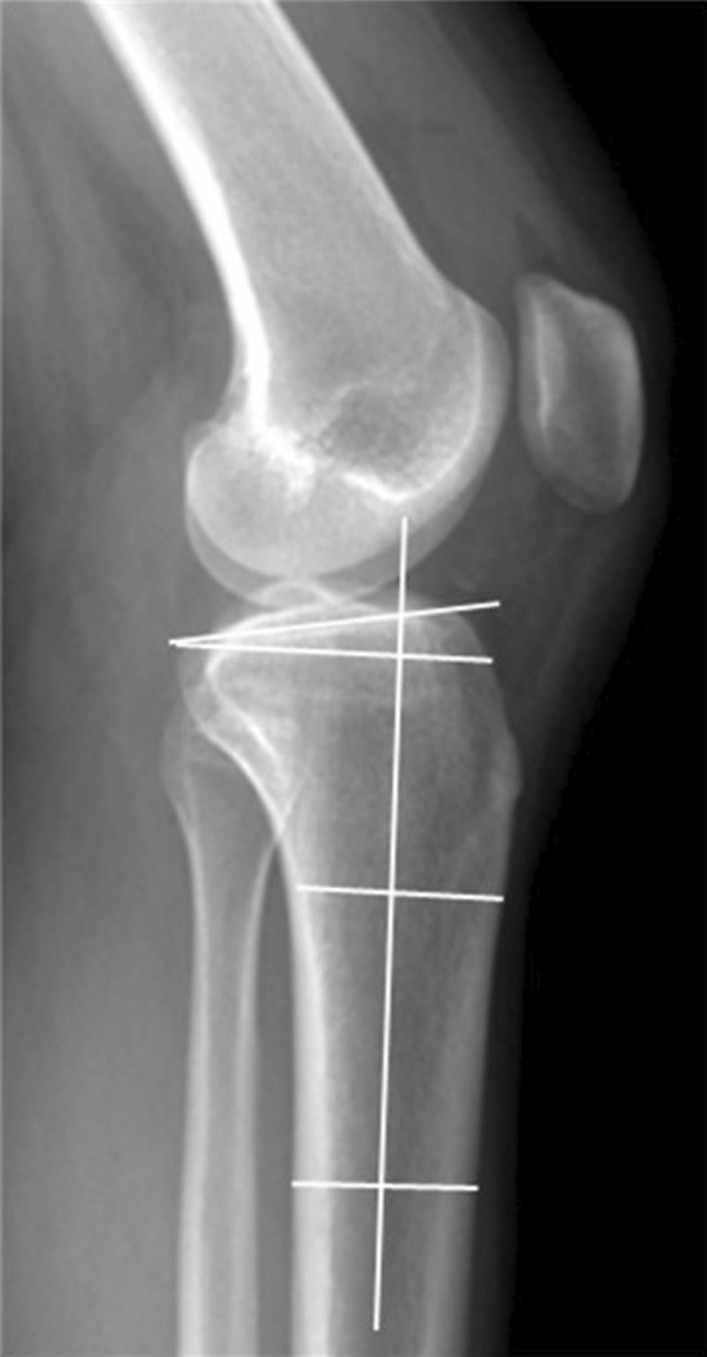
Measurement of the posterior tibial slope. The posterior tibial slope was defined as the angle between the line connecting the anterior and posterior aspects of the tibial plateau, and the line perpendicular to the anatomical axis of the tibia

Morphological measurements in radiographs included lateral femoral condyle ratio (LFCR), condyle flexion angle (CFA), and posterior tibial slope (PTS). Two separate circles with a distance of 5 cm were determined in the center of the femoral shaft. The more distal one was positioned at the most proximal aspect of the trochlea. The line passing through the centers of the two circles was determined to identify the long axis of the distal femur. The line between the most posterior point and most anterior point of the lateral condyle was determined to identify the axis of the lateral femoral condyle. The LFCR was defined as the ratio of the distance from the intersection of the two axes to the most posterior point of the lateral condyle, to the total anteroposterior distance of the lateral condyle (Fig. 3) [23]. The CFA was defined as the angle between the long axis of the distal femur and the axis of the lateral femoral condyle (Fig. 3) [23]. The PTS was defined as the angle between the line connecting the anterior and posterior aspects of the tibial plateau, and the line perpendicular to the anatomical axis of the tibia connecting the centers of the tibial shaft at two positions (Fig. 4) [24].

### Morphology of the ACL

**Fig. 5:**
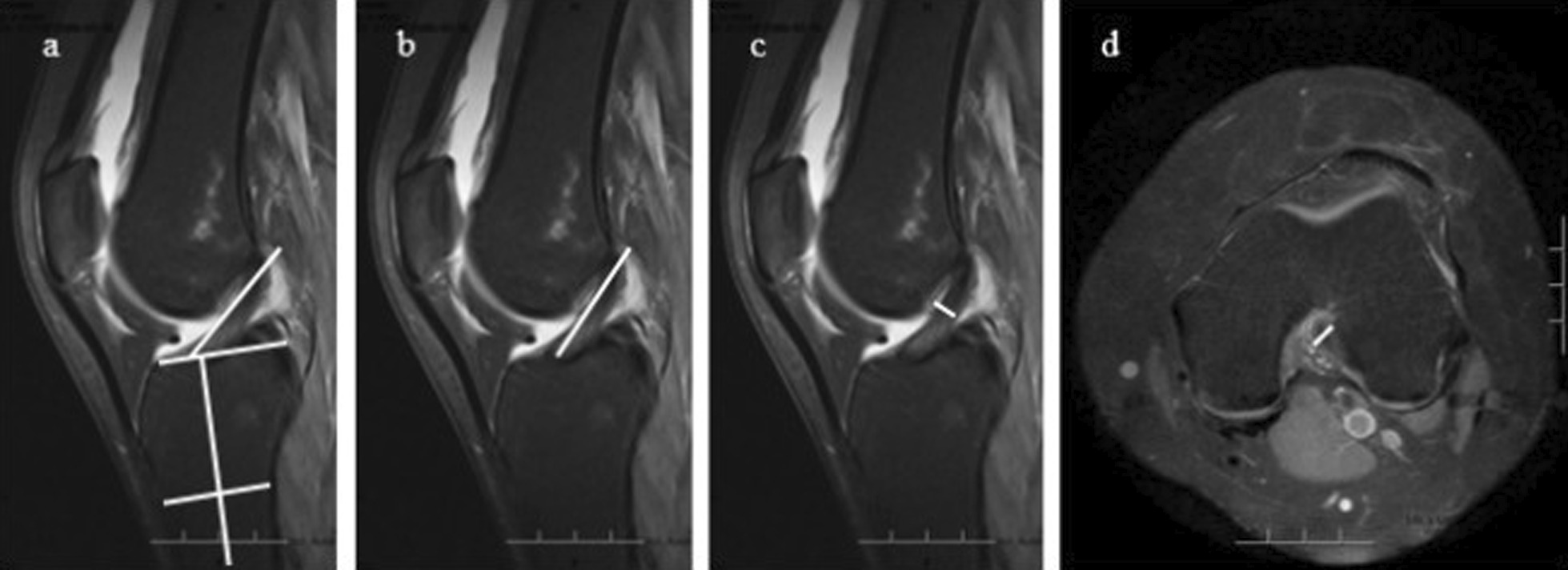
Measurements of the ACL in the sagittal **a**–**c** and axial **d** planes. The inclination angle **a** was formed between the line perpendicular to the anatomical axis of the tibia and the most anterior fibers of the ACL. The ACL length **b** was the distance between the tibial midpoint and the femoral midpoint of the ACL attachments. The maximal anteroposterior thickness of the ACL **c** was evaluated at the outlet of the intercondylar notch on the sagittal view. The maximal transverse width **d** was evaluated on the axial view showing the widest ACL. *ACL* Anterior cruciate ligament

Morphological measurements of the ACL in MRI included ACL length, inclination angle, and ACL size. On the sagittal view, the slice that best displayed the intra-articular course of the ACL was chosen for measurements of the ACL length and inclination angle, as described by van Diek et al. [25]. The inclination angle was formed between the most anterior fibers of the ACL and the line perpendicular to the anatomical axis of the tibia (Fig. 5a). The ACL length was the distance between the tibial midpoint and the femoral midpoint of the ACL attachments (Fig. 5b).

The ACL size was measured as Anderson et al. [26] described. The maximal anteroposterior thickness of the ACL was evaluated at the outlet of the intercondylar notch in the sagittal plane, which was identified by the anterior limit of Blumensaat’s line (Fig. 5c). The maximal transverse width was evaluated in the axial plane showing the widest ACL (Fig. 5d). The ACL size was measured perpendicular to the long axis of the ligament fibers.

### Statistical analysis

Measurement data were expressed as the means ± standard deviations for continuous variables, and counts for categorical variables. The Levene’s test and Kolmogorov–Smirnov test were used to check the homogeneity and normality of the data. All numerical variables showed a normal distribution and equal variance. The independent samples *t*-test was performed to examine differences in continuous variables. The chi-square test was used for comparing categorical variables. The data were analyzed using IBM SPSS version 21.0 (Statistical Package for the Social Sciences, Inc.; Chicago, IL). Differences were considered significant at *p* < 0.05.

The minimum sample size was calculated with an a priori power calculation using G*Power version 3.1.9.4 (Heinrich-Heine-Universitat Dusseldorf, Dusseldorf, Germany). To achieve a power of 80% and a two-sided significance level of 0.05, a minimum of 34 samples per group was needed to detect a 1 mm notch width difference.

## Results

In total, 240 patients were included: 80 patients in PI group, including 28 males and 52 females, with the average age of 24.1 ± 10.2 years; and 160 patients in control group, including 56 males and 104 females, with the average age of 24.7 ± 7.5 years. There was no significant difference regarding demographic information between PI group and control group (Table 1).

**Table 1:** Patient demographics in patellar instability group and control group

Variable	Patellar instability group	Control group	*P* value
Number of patients, *n*	80	160	–
Number of knees, *n*	80	160	–
Sex (male: female), *n*	28/52	56/104	–
Side (left: right), *n*	42/38	90/70	0.702
Age, y	24.1 ± 10.2	24.7 ± 7.5	0.701
Body mass index, kg/m^2^	23.2 ± 3.3	22.7 ± 3.2	0.495

Values are reported as means ± standard deviations unless noted otherwise

### Measurement reliability

The ICC values of all measurement parameters were good to excellent, with the intraobserver ICCs ranging from 0.831 to 0.935, and the interobserver ICCs ranging from 0.809 to 0.924, which demonstrated a strong intra- and interobserver reliability for all measurements.

### Comparisons between PI group and control group

**Fig. 6:**
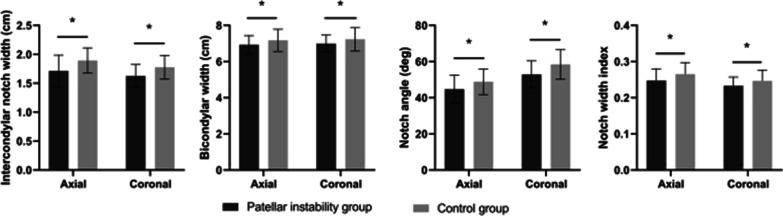
Comparisons of the femoral condyles between patellar instability group and control group. Intercondylar notch width, bicondylar width, notch angle, and notch width index in both axial and coronal planes were significantly smaller in patellar instability group than those of control group (*p* < 0.05). ^∗^*p* < 0.05 was deemed statistically significant

The morphological parameters of the femoral condyles between PI group and control group are shown in Table 2. The intercondylar notch width, bicondylar width, notch width index, and notch angle in both axial and coronal planes were significantly smaller in PI group than those of control group (*p* < 0.05), which revealed that patients with PI had a narrow intercondylar notch (Fig. 6). The medial condylar width and lateral condylar width were smaller in PI group, but the differences did not reach the statistical level (*p* > 0.05). The LFCR was significantly larger in PI group than that of control group (*p* = 0.018). No other dimensional or angular measurements had significant differences between the two groups.

**Table 2:** Morphological parameters of the femoral condyles between patellar instability group and control group

Variable	Patellar instability group	Control group	*P* value
Notch height, cm			
Axial	2.94 ± 0.34	3.04 ± 0.38	0.165
Coronal	2.37 ± 0.27	2.37 ± 0.25	0.968
Notch width, cm			
Axial	1.71 ± 0.27	1.89 ± 0.22	< 0.001^*^
Coronal	1.63 ± 0.20	1.78 ± 0.21	< 0.001^*^
Medial condylar width, cm			
Axial	2.51 ± 0.24	2.57 ± 0.31	0.299
Coronal	2.49 ± 0.21	2.57 ± 0.29	0.135
Lateral condylar width, cm			
Axial	2.70 ± 0.25	2.71 ± 0.31	0.870
Coronal	2.88 ± 0.29	2.89 ± 0.37	0.803
Bicondylar width, cm			
Axial	6.93 ± 0.51	7.18 ± 0.62	0.030^*^
Coronal	6.99 ± 0.48	7.24 ± 0.65	0.036^*^
Notch width index			
Axial	0.25 ± 0.03	0.27 ± 0.03	0.005^*^
Coronal	0.23 ± 0.02	0.25 ± 0.03	0.016^*^
Notch angle, deg			
Axial	44.76 ± 7.73	48.89 ± 7.11	0.004^*^
Coronal	52.92 ± 7.47	58.47 ± 8.21	< 0.001^*^
Lateral femoral condyle ratio	0.68 ± 0.05	0.66 ± 0.04	0.018^*^
Condyle flexion angle, deg	88.84 ± 6.60	90.55 ± 6.39	0.176
Posterior tibial slope, deg	9.84 ± 3.88	11.03 ± 4.02	0.128

Values are reported as means ± standard deviations

^*^p < 0.05 was considered significant

**Fig. 7:**
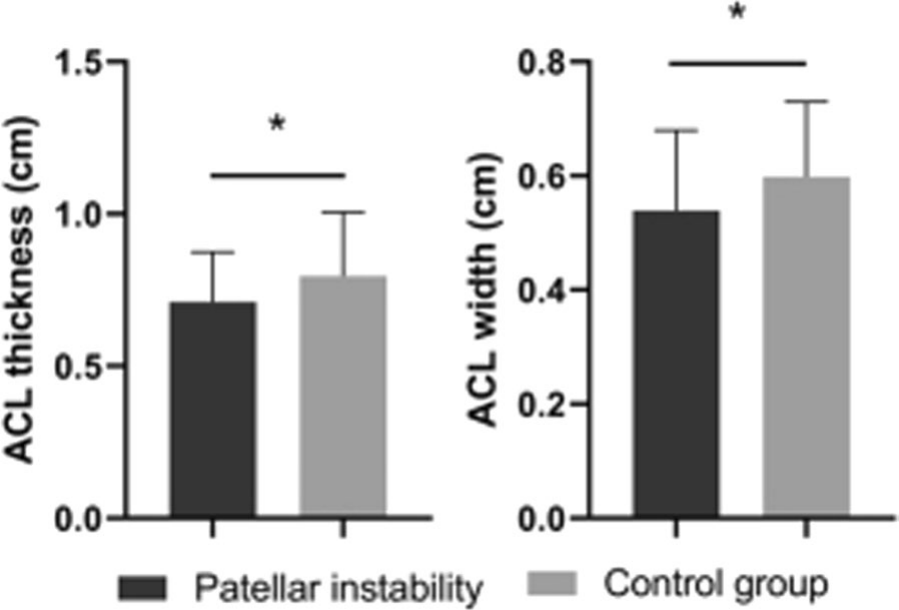
Comparisons of the ACL between patellar instability group and control group. The ACL thickness and width were significantly smaller in patellar instability group than those of control group (*p* < 0.05). ^∗^*p* < 0.05 was deemed statistically significant. *ACL* Anterior cruciate ligament

The morphological parameters of the ACL between PI group and control group are shown in Table 3. The anteroposterior thickness of the ACL in the sagittal plane and transverse width in the axial plane were significantly smaller in PI group than those of control group (*p* < 0.05) (Fig. 7), while there was no significant difference in the ACL length and inclination angle between the two groups (*p* > 0.05).

**Table 3:** Morphological parameters of the ACL between patellar instability group and control group

Variable	Patellar instability group	Control group	*P* value
Inclination angle, deg	49.40 ± 6.24	47.78 ± 7.83	0.258
ACL length, cm	3.79 ± 0.33	3.84 ± 0.37	0.497
ACL thickness, cm	0.70 ± 0.16	0.80 ± 0.21	0.023^*^
ACL width, cm	0.54 ± 0.14	0.60 ± 0.13	0.029^*^

Values are reported as means ± standard deviations. *ACL* Anterior cruciate ligament

^*^p < 0.05 was considered significant

### Comparisons between male and female patients in PI group

The sex comparison was performed in PI group. The male patients had an average age of 25.1 ± 10.2 years and an average body mass index (BMI) of 23.7 ± 3.1, and the female patients had an average age of 23.5 ± 10.3 years and an average BMI of 22.9 ± 3.5. There was no significant difference in the age, BMI, and laterality between male and female patients.

The morphological parameters of the femoral condyles are shown in Table 4. Notch width, medial condylar width, lateral condylar width, bicondylar width in both axial and coronal planes were significantly smaller in female patients than those of male patients (*p* < 0.05). However, when the notch width was normalized by the bicondylar width, no significant difference was observed in the notch width index in either axial or coronal planes (*p* > 0.05). The morphological parameters of the ACL were shown in Table 5, with no significant difference between male and female patients (*p* > 0.05).

**Table 4:** Morphological parameters of the femoral condyles between male and female patients in patellar instability group

Variable	Male	Female	*P* Value
Notch height, cm			
Axial	3.00 ± 0.38	2.90 ± 0.31	0.416
Coronal	2.46 ± 0.24	2.33 ± 0.27	0.138
Notch width, cm			
Axial	1.88 ± 0.34	1.63 ± 0.18	0.005^*^
Coronal	1.73 ± 0.25	1.57 ± 0.14	0.013^*^
Medial condylar width, cm			
Axial	2.66 ± 0.21	2.44 ± 0.22	0.004^*^
Coronal	2.64 ± 0.21	2.40 ± 0.15	< 0.001^*^
Lateral condylar width, cm			
Axial	2.91 ± 0.24	2.59 ± 0.18	< 0.001^*^
Coronal	3.04 ± 0.32	2.79 ± 0.24	0.007^*^
Bicondylar width, cm			
Axial	7.44 ± 0.33	6.65 ± 0.34	< 0.001^*^
Coronal	7.41 ± 0.49	6.76 ± 0.27	< 0.001^*^
Notch width index			
Axial	0.25 ± 0.04	0.25 ± 0.03	0.511
Coronal	0.23 ± 0.03	0.23 ± 0.02	0.915
Notch angle, deg			
Axial	46.93 ± 10.19	43.58 ± 5.92	0.195
Coronal	55.51 ± 7.58	51.52 ± 7.17	0.108
Lateral femoral condyle ratio	0.68 ± 0.05	0.69 ± 0.05	0.420
Condyle flexion angle, deg	90.32 ± 7.68	88.05 ± 5.96	0.305
Posterior tibial slope, deg	9.83 ± 3.20	9.85 ± 4.26	0.987

Values are reported as means ± standard deviations

^*^p < 0.05 was considered significant

**Table 5:** Morphological parameters of the ACL between male and female patients in patellar instability group

Variable	Male	Female	*P* value
Inclination angle, deg	48.61 ± 6.24	49.82 ± 6.32	0.563
ACL length, cm	3.88 ± 0.31	3.75 ± 0.33	0.203
ACL thickness, cm	0.69 ± 0.18	0.72 ± 0.16	0.474
ACL width, cm	0.51 ± 0.10	0.55 ± 0.16	0.344

Values are reported as means ± standard deviations. *ACL* Anterior cruciate ligament

## Discussion

The most important finding of this study was that the morphology of the intercondylar notch and ACL in patients with PI differed significantly from controls, demonstrating that patients with PI had a stenotic intercondylar notch and a thin ACL. Notch width was significantly smaller in female patients than that of males, but no sex difference regarding the notch width index and morphology of the ACL was found. When ACL reconstruction is performed for patients with PI, the differences in the intercondylar notch and ACL should be taken into consideration.

Acceptance of the “individualized ACL reconstruction” concept and rejection of the “one size fits all” belief during the last decade have motivated surgeons to investigate the effect of patient-specific anatomical and morphological characteristics on the outcomes after ACL reconstruction, and how can ACL reconstruction be performed specifically based on patient’s individualized anatomy and lifestyle [2, 3, 27]. There is variation in the morphology of the intercondylar notch and ACL between individuals, including intercondylar notch size, ACL insertion site size, and ACL length, which should be considered during ACL reconstruction to guide the selection of the surgical technique, graft option, graft diameter, and tunnel size [2, 28].

Intercondylar notch is one of the most noticeable anatomical factors in ACL reconstruction, which varies considerably in the shape, size, and orientation between individuals. More recent studies have demonstrated that ACL failure or re-injury was correlated with a smaller notch width considering the close physical contact between the notch and ACL [11, 29]. The present study investigated morphological characteristics of the intercondylar notch in cases of PI, showing that a stenotic intercondylar notch was present in patients with PI. Therefore, this abnormality should be taken into account when choosing the graft size to reduce the risk of failure. If a normal or oversized ACL graft is selected, the graft is actually thicker in a stenotic notch, which can increase strain on the ACL, and produce impingement on the lateral femoral condyle or posterior cruciate ligament (PCL) during knee range of motion [30, 31]. Furthermore, a narrow notch limited the range of motion of the ACL, followed by increased possibility of collision between the graft and the notch [22]. Continuous graft impingement can lead to graft deterioration or re-injury, range of motion deficit, and even knee instability [31, 32].

The LFCR that indicates posterior femoral condylar depth was significantly larger in patients with PI, which is a risk factor for ACL injury [23]. Increased LFCR could lead to altered knee kinematics, gait, and load mechanism [33]. Furthermore, increased LFCR could result in longer lateral and anterolateral knee structures, such as lateral collateral ligament and anterolateral aspect of the capsule, and thus larger anisometry in flexion, which affects knee rotatory stability [23].

The abnormal morphology of the intercondylar notch in patients with PI may come from the abnormalities of the femoral condyles, and torsional deformity of the lower limb. The morphology of the intercondylar notch is shaped by medial and lateral femoral condyles. Patients with PI often have different morphological characteristics at both anterior and posterior parts of the femoral condyles. A previous study reported that 96% of patients with PI had trochlear dysplasia, which is an abnormality of the shape and depth of the trochlear groove [34, 35]. Isıklar et al. [36] observed that mild trochlear dysplasia caused intercondylar notch stenosis instead of changes of the patellar localization, indicating that dysplasia in the anterior distal femur may cause notch stenosis in the posterior distal femur. Patients with trochlear dysplasia also had different posterior femoral condyles, characterized by a smaller lateral posterior condyle and a bigger medial posterior condyle [18]. In addition, the distal femur may rotate internally because of condylar abnormalities during axial torsion, which can lead to a valgus angle, transfer the force center to the lateral condyle, and finally increase the stress on the ACL [37].

As ACL reconstruction is a kind of graft transplantation, it is important to assess the size of the graft in detail, and determine the size as close as that of the native ACL. However, in most cases of ACL reconstruction, the graft size is determined mainly by the harvested graft size [38]. Risk of failure will increase if surgeons determine the reconstructed ACL size only by harvested graft size [39]. Therefore, the size of the ACL was also measured in this study, showing that the anteroposterior thickness and transverse width of the ACL were significantly smaller in patients with PI. If the graft of the same size as that of the normal patients is used in patients with PI, the expected outcomes may not be obtained due to the mismatch between the size of the graft and notch. If there is more mismatch between graft and notch size, impingement may occur [40, 41]. This was demonstrated by Nishimori et al. [42] who reported that in cases of ACL reconstruction with impingement on the PCL, the graft was thick, while in cases without impingement, the graft was thin. Therefore, the graft used in ACL reconstruction should bear more resemblance to the native ACL.

In order to perform accurate anatomical ACL reconstruction, it might be crucial to predict the size of the ACL by measuring morphological parameters of the intercondylar notch and ACL prior to graft selection. There is a significant correlation between the intercondylar notch width and the ACL width [43]. Patients with a smaller intercondylar notch usually had a relatively smaller and weaker ACL [44, 45]. This study also confirmed that patients with PI had a narrower intercondylar notch and a corresponding thinner ACL. Therefore, preoperative measurement may be an easy and effective way to evaluate the native ACL size, and direct surgeons to select the most suitable graft type and size. Surgeons should be careful to place the graft anatomically to decrease the risk failure of ACL reconstruction and not to overfill the intercondylar notch [2]. In addition, impingement of the graft on the intercondylar notch and the PCL should be avoided [11, 31]. Bone tendon healing and graft remodeling may be affected by the communication between the bone tunnels due to inappropriate graft movements [45, 46].

Regarding sex comparisons of patients with PI, the intercondylar notch width was significantly smaller in female patients than that of male patients, but no significant difference was observed when the notch width was standardized by the bicondylar width. This difference may be due to that the size of the knee in female is smaller than male. Therefore, considering no sex difference in the notch width index and notch angle, the extent of intercondylar notch stenosis was similar between males and females, which was consistent with that no significant difference in the ACL size was observed between genders.

This study is clinically relevant in that by measuring morphological parameters of the intercondylar notch and ACL, surgeons can better understand the native ACL, and further individualize ACL reconstruction and design better procedures for patients with PI to improve patient outcomes after ACL reconstruction. The results of this study may be useful in graft size selection to reproduce the native anatomy of ACL as much as possible in cases of PI. When observing that the intercondylar notch is narrow, surgeons would adopt corresponding surgical techniques, such as changing the type of graft, and performing a plasty of the same. As the strong point of this study, the measurement methodology had a high accuracy and reproducibility.

This study has certain limitations. First, arthroscopic measurement with more accuracy was not performed due to its retrospective nature. Second, the difference with respect to age was not included. PI is common in children and adolescents, resulting in an uneven age distribution across patients. Third, the proportion of each sex was not equal, and more females were included than males. However, PI and ACL injury are more common in females. Fourth, the subjects in the control group were not completely healthy individuals, but they were carefully screened and excluded if there were any signs to the knee other than isolated meniscus injury. Fifth, MRI for outlining bony structures from surrounding tissues has limitations, but it has more advantages in displaying cartilage to identify the borders of the intercondylar notch. Sixth, establishing an exact geometric relationship between the intercondylar notch and ACL size was difficult, because the ACL is a three-dimensional helicoid structure. Seventh, this study did not show how many patients suffered from ACL injuries in PI group and control group, and thus the relationship between PI and the risk of ACL injury cannot be obtained. The patient number in this study met statistical requirement of minimum sample size, but further clinical research with a larger sample size is still required to confirm our findings.

## Conclusion

The patient with PI had a stenotic intercondylar notch and a thin ACL. No significant sex difference in the intercondylar notch stenosis and ACL size was observed. The morphology of the intercondylar notch and ACL should be taken into consideration when planning individualized ACL reconstruction in the presence of PI.

## Data Availability

The datasets used and analyzed during the current study are available from the corresponding author on reasonable request.

## Abbreviations

ACL: Anterior cruciate ligament
CFA: Condyle flexion angle
ICC: Intraclass correlation coefficient
LFCR: Lateral femoral condyle ratio
MRI: Magnetic resonance image
PCL: Posterior cruciate ligament
PI: Patellar instability
PTS: Posterior tibial slope
T1WI: T1 weighted image
T2WI: T2 weighted image
